# Newly Emerging *Streptococcus salivarius* G7 as a Probiotic Candidate for Oral Health

**DOI:** 10.3390/microorganisms14061234

**Published:** 2026-05-30

**Authors:** Insoon Chang, Sung-Hoon Lee

**Affiliations:** 1 Division of Regenerative and Reconstructive Sciences, School of Dentistry, University of California Los Angeles, Los Angeles, CA 90095, USA; ichang@dentistry.ucla.edu; 2 Department of Oral Microbiology and Immunology, College of Dentistry, Dankook University, Cheonan 31116, Republic of Korea

**Keywords:** *S. salivarius* G7, probiotics, oral biofilm, oral probiotics

## Abstract

Probiotics are known to benefit human health through improving the gut environment. This study aimed to investigate whether *Streptococcus salivarius* (*S. salivarius*) G7 exhibits probiotic properties and evaluated its effectiveness and suitability for oral health applications. Whole-genome sequencing of *S. salivarius* was performed using Novo assembly and bioinformatics analysis. To determine probiotic suitability, the required metabolic profiles were obtained through performing a hemolysis test, antibiotic susceptibility test, D-lactate production assay, and cytotoxicity assay according to the methods recommended in World Health Organization guidelines. To investigate the oral health impacts of *S. salivarius*, the acidogenicity and antimicrobial activity of *S. salivarius* were investigated. Finally, oral biofilms treated and untreated with *S. salivarius* were investigated. The phylogenetic and bioinformatic analyses confirmed the taxonomic identity of *S. salivarius*. Also, it has been proven that this bacterium carries no virulence factors or transmissible antibiotic resistance genes. *S. salivarius* G7 exhibited low antibiotic resistance, cytotoxicity, and acidogenicity, while also displaying antimicrobial activity against oral disease-related bacteria, and was able to maintain eubiosis in oral biofilms. *S. salivarius* G7 met all the safety assessment criteria required by current probiotic guidelines and exhibited beneficial properties for oral health. Therefore, this strain may represent a safe and promising probiotic candidate for oral health applications.

## 1. Introduction

The term *probiotics* has evolved considerably over the past four decades. Initially, it referred to substances produced by prokaryotes that stimulated the growth of other prokaryotes [1]. The most widely accepted contemporary definition, proposed by the Food and Agriculture Organization and the World Health Organization (FAO/WHO) in 2001, describes probiotics as “live organisms which, when administered in adequate amounts, confer a health benefit on the host” [2]. Probiotics development has largely followed this definition and the associated FAO/WHO guidelines, with most formulations designed to enhance the microbial environment of the intestinal tract using bacteria of intestinal origin. More recently, recognizing the distinct ecological and physiological characteristics of the oral cavity, attention has shifted toward evaluating and formulating probiotics specifically tailored for oral health applications [3].

*Streptococcus salivarius* (*S. salivarius*) is one of the earliest colonizers of the human oral cavity [4] and plays important ecological roles by forming a protective barrier against pathogenic microorganisms and reducing their adhesion and colonization [5]. In particular, *S. salivarius* exerts antagonistic effects against group A streptococci and other oral pathogens through the production of bacteriocin-like inhibitory substances [5,6]. To enhance its competitiveness within the oral ecosystem, *S. salivarius* synthesizes class I bacteriocins (salivaricin 9, salivaricin A, salivaricin B, salivaricin D, salivaricin E, and salivaricin G32) and class II bacteriocins (salivaricin V, salivaricin W, salivaricin X, salivaricin Y, salivaricin Z, and bacteriocin-like peptide) [7,8,9]. These salivaricins are characterized by the presence of unusual amino acids such as lanthionine and methyllanthionine [10]. Due to these properties, certain *S. salivarius* strains have been developed as probiotics intended for oral health applications [11,12,13].

Infectious diseases of the oral cavity can be broadly categorized into two major groups: gingival and dental diseases. Prior studies have primarily focused on elucidating the relationship between the presence and virulence of pathogenic bacteria and the onset of oral infections, as well as strategies for bacterial elimination. Recently, interests have shifted toward understanding the relationship between the oral microbial environment, bacterial distribution, and oral disease development [14]. Oral infectious diseases are closely associated with oral biofilms, which consist of complex, multispecies microbial communities [15]. In healthy individuals, these biofilms maintain a balanced composition dominated by commensal bacteria. However, when this balance is disrupted, due to changes in the oral environment, certain pathogenic species may increase in abundance, resulting in a higher proportion of disease-associated bacteria [16]. This microbial imbalance ultimately leads to oral disease [16]. Furthermore, it has been proposed that such bacterial dysbiosis could be restored to a symbiotic, healthy state if the environmental disturbances are corrected [16].

In this study, we aimed to investigate whether *S. salivarius* G7 exhibits probiotic properties and evaluate its beneficial effects on oral health.

## 2. Materials and Methods

### 2.1. Bacterial Strains and Culture Conditions

*S. salivarius* G7 (formerly denoted as KCOM 2137) was isolated from the oral cavity of a healthy male adult (Korean collection of type culture) and aerobically maintained in tryptic soy broth (TSB; BD Biosciences, Sparks, MD, USA). *Streptococcus salivarius* ATCC 7073 (type strain), *Streptococcus mutans* (*S. mutans*) ATCC 25175, and *Enterococcus faecalis* (*E. faecalis*) ATCC 29212 were cultured using TSB. *Aggregatibacter actinomycetemcomitans* (*A. actinomycetemcomitans*) ATCC 43178 was cultivated using brain heart infusion (BHI; BD Biosciences, Sparks, MD, USA), and *Porphyromonas gingivalis* (*P. gingivalis*) ATCC 33277 was cultivated using BHI supplemented with vitamin K (0.2 mg/mL) (Sigma-Aldrich, San Jose, CA, USA) and hemin (5 mg/mL) (Sigma-Aldrich) at 37 °C under anaerobic conditions (5% H_2_, 10% CO_2_, 85% N_2_).

### 2.2. Phylogenetic Analysis

Whole-genome sequencing (WGS) of *S. salivarius* G7 was performed by Macrogen Inc. (Seoul, Republic of Korea). Briefly, the whole genome was sequenced using a 150 bp pair-end library with the Illumina HiSeq 2500 system (Illumina Inc., San Diego, CA, USA), and the Illumina short reads were subjected to adapter trimming and quality control with Trimmomatic ver. 0.36. The short reads were assembled using Platanus-allee 2.2.2 bioinformatics software [17], with error correction performed with PBjelly 2 software from PBsuit (v15.8.24). After assembly, an accurate genomic sequence was obtained from the draft sequence using Pilon ver 1.23, and then the consensus sequence was generated. The genome size of the sample was estimated by K-mer analysis, and the complete genome was annotated. Ultimately, 16S rRNA sequences were obtained from the full *S. salivarius* G7 genome, and genes of other bacterial strains were obtained via IMG (http://img.jgi.doe.gov/, accessed on 1 May 2022). Phylogenies were reconstructed using the Bayesian and Maximum Likelihood methods in MEGA 12 [18].

### 2.3. Genomic Analysis for Antibiotic Resistance and Bacteriocin Genes

In order to identify antibiotic resistance genes in the *S. salivarius* G7 genome, the Comprehensive Antibiotic Resistance Database (CARD; https://card.mcmaster.ca/ accessed on 1 May 2022) was employed [19]. The whole genomic sequences were input into the Resistance Gene Identifier (RGI) tool on the CARD website, and were screened for bacteriocin genes using BAGLE4 (http://bagel4.molgenrug.nl/ accessed on 15 June 2022). The search results with a bit score of 50 or higher were selected, and tBLASTn 2.15.0 was used to confirm the bacteriocin’s presence and compare it with other *S. salivarius* strains.

### 2.4. Metabolic Profile

API 50 CH and API 20 strip kits (BioMérieux, Bruz Cédex, France) were used to analyze the metabolic profile of *S. salivarius* G7 using the manufacturer’s recommended method. Briefly, *S. salivarius* G7 was cultivated anaerobically at 37 °C overnight and washed three times with phosphate-buffered saline (PBS, pH 7.2). The bacterial concentration was adjusted to 1.0 O.D. at a wavelength of 660 nm (as a level of 4 McFarland) with PBS, and then the bacterial suspension was inoculated into API 50 CHB/E and API NaCl 0.85% media. After inoculating the media into each well of the API 50 CH and API 20 streps, both kits were incubated at 37 °C for 24 h aerobically, and then the fermentation profiles were analyzed.

### 2.5. Hemolytic Activity

The hemolytic activity of *S. salivarius* G7 was analyzed by streaking the bacteria onto blood agar composed of blood agar base (BD Biosciences, Sparks, MD, USA) and 5% (vol/vol) sterile defibrinated sheep blood (Kisan Bio, Seoul, Republic of Korea; Cat No. MB-S1876). The plate was incubated at 37 °C for 24 h, and the color change in the area surrounding the bacteria was observed.

### 2.6. Antibiotic Susceptibility Test

The sensitivity of *S. salivarius* G7 to antibiotics was examined according to methods recommended by the International Organization for Standardization (ISO) 10932:2010 standard and ETEST [20,21]. For the susceptibility test, antibiotics related to a wide range of resistance determinants (ampicillin, chloramphenicol, clindamycin, erythromycin, gentamycin, kanamycin, streptomycin, tetracycline, and vancomycin) were selected based on the European Food Safety Authority (EFSA) guidelines [22]. The bacterial suspensions were adjusted to 0.5 MacFarland with sterile saline, and the prepared bacterial suspension was spread with a sterile swab onto Mueller–Hinton agar supplemented with 5% sheep blood and 20 mg/L of beta-NAD. The antibiotic disks were placed onto the bacteria-inoculated agar, and the plates were incubated at 37 °C for 48 h under anaerobic conditions. Then, the clear zone was measured with a vernier caliper. In another experiment, the ETEST was performed using a predefined gradient of antibiotic concentrations on a plastic strip (E-TEST^®^, Biomèrieux, Marcy I`Ètoile, France) to determine the minimum inhibitory concentrations (MICs) of the antibiotics.

### 2.7. D-Lactate Production Assay

*S. salivarius* G7 was cultivated in TSB supplemented with 0.5% glucose at 37 °C for 24 h under anaerobic conditions (5% H_2_, 10% CO_2_, and 85% N_2_). The cultured medium was collected by centrifuging at 9000× *g* for 10 min and then heated at 80 °C for 15 min to stop the enzymatic reaction. The prepared sample’s D-lactate levels were measured using a D-lactate assay kit (Abcam Co., Cambridge, UK).

### 2.8. Cytotoxicity Assay

In order to analyze the cytotoxicity of *S. salivarius* G7, YD-38, an oral cancer cell line, and HT-29, a colon cancer cell line, were used. The cells were maintained in Dulbecco’s modified Eagle’s medium (DMEM; Hyclone, Logan, UT, USA) supplemented with 10% fetal bovine serum (Hyclone) and 100 U/mL of penicillin–streptomycin (Hyclone). The cells (1 × 10^4^ cells/well) were seeded into a 24-well plate (SPL Biosciences, G) and cultivated at 37 °C in a 5% CO_2_ atmosphere for 18 h. After replacing old cell culture media with fresh media without antibiotics, *S. salivarius* G7 (1 × 10^7^, 1 × 10^8,^ and 1 × 10^9^ cells/well) was inoculated into the wells, and the plates were incubated for 26 h in a CO_2_ incubator (Thermo Scientific, Waltham, MA, USA). The cytotoxicity of *S. salivarius* G7 was investigated by measuring lactate dehydrogenase (LDH) using the Cytotoxicity LDH assay kit (Dojindo, Kyoto, Japan). As a comparison group, the cells were treated with *Porphyromonas gingivalis* ATCC 33277 and *Escherichia coli* ATCC 35150 at the same level as *S. salivarius* G7. Also, 2% and 4% Triton X-100 (Sigma-Aldrich, San Jose, CA, USA) in DMEM for HT-29 and YD-38 were used as positive controls.

### 2.9. Investigation of Acidogenicity

To investigate acidogenicity, *S. salivarius* and *S. mutans* (1 × 10^8^ cells/mL) were inoculated into 100 mL of TSB or TSB with 2% sucrose and then incubated at 37 °C anaerobically. The bacterial suspension was collected every 2 h, and the pH of the suspension was measured using a pH meter (Thermo Fisher Scientific, Waltham, MA, USA). pH measurements were taken over 16 h. As a comparison group, the cells were treated with *S. mutans* ATCC 25175 at the same level as *S. salivarius* G7.

### 2.10. Antimicrobial Activity Assay

To investigate the production of antimicrobial substances by *S. salivarius* G7, a susceptibility assay was conducted using the supernatant of *S. salivarius* conditioned medium (SCM). The antimicrobial activity of SCM was assessed employing the methods provided by the Clinical Laboratory Standard Institute (CLSI). SCM (180 μL) was added to the 12th row of the wells containing TSB. Two-fold serial dilutions were conducted with a micropipette. After adjusting the *S. mutans* and *E. faecalis* concentration with 0.5 McFarland suspension, 20 μL of the bacterial suspension was inoculated into the prepared well, and the plate was incubated at 37 °C for 24 h under anaerobic conditions (5% H_2_, 10% CO_2_, and 85% N_2_). In another experiment, the antimicrobial activity of SCM against periodontitis-related bacteria was evaluated. SCM (180 μL) was added to the 12th row of the wells containing BHI broth for *A. actinomycetemcomitans* and BHI broth including hemin and vitamin K for *P. gingivalis*. Two-fold serial dilutions were conducted with a micropipette. After adjusting the bacterial concentration with 0.5 McFarland suspension, 20 μL of the bacterial suspension was inoculated into the prepared well, and the plate was anaerobically incubated at 37 °C for 36 h. The growth of bacteria was measured as optical density (660 nm wavelength) using a spectrophotometer (Biotek, Winooski, VT, USA).

### 2.11. Observation of Oral Biofilm

Biofilm formation experiments were conducted with an in vitro experimental model using salivary bacteria. Saliva samples were collected from five men and five women aged 20 to 25 who were following a standard Korean diet without any particular food bias and agreed to participate in the experiment. The experimental procedure was approved by the Institutional Review Board (IRB; Approval No. 2024-10-060-005), and informed consent was obtained from all participants for the use of human-derived materials. Unstimulated saliva was collected from 10 healthy people, and the pooled saliva was mixed with the same volume of BHI broth. In order to remove debris, the supernatant was transferred into a new tube after centrifugation at 2000× *g* for 10 min at 4 °C. The prepared bacterial suspension was added along with 2% sucrose and *S. mutans* (1 × 10^8^ cells/mL) to form a cariogenic biofilm and then dispensed into a 12-well plate (SPL Lifescience) and an 8-well glass chamber (BD Falcon, Franklin Lakes, NJ, USA). The prepared plate and chamber were incubated for 7 days, during which the cariogenic biofilm formed. The biofilm was treated with SCM at various time points. In another experiment, when firming the biofilm, *S. salivarius* G7 was added to the prepared plate and chamber every day. The plate and chamber were incubated anaerobically at 37 °C for 7 days with the medium (BHI including 2% sucrose) changed every day; then, *S. salivarius* G7 was added. The biofilm was washed twice with PBS. For the bacterial count, the biofilm on a 12-well plate was washed twice with PBS to remove planktonic bacteria, and 1 mL of BHI broth was added. The biofilm was mechanically disrupted with a scraper (Corning Co., Corning, NY, USA) and transferred into a 1.5 mL tube, followed by homogenization using a vortex. The suspension was serially diluted with fresh TSB broth, and the diluted bacterial suspensions were inoculated on BHI, Mitis-salivarius (MS), and Mitis-salivarius bacitracin (MSB) agar plates (BD Bioscience). The plates were incubated at 37 °C for 72 h, and the colonies (colony-forming unit, CFU) of total bacteria, oral streptococci, and *S. mutans* were counted. Also, to observe the biofilm in an 8-well glass chamber, it was washed twice with PBS and stained using a bacterial live/dead staining kit (Invitrogen, Waltham, MA, USA); then, it was observed using an LSM 700 confocal laser scanning microscope (Carl-Zeiss, Oberkochen, Germany).

### 2.12. Statistical Analysis

IBM SPSS Statistics version. 30 (IBM, Armonk, NY, USA) was used for statistical analysis. Data distribution was evaluated using the Kolmogorov–Smirnov test. The differences between the two independent groups were analyzed by the Mann–Whitney U test, and the differences with *p* values less than 0.05 were considered statistically significant. The values are expressed as the median and interquartile range.

## 3. Results

### 3.1. Whole Genomic Sequence Analysis

**Figure 1:**
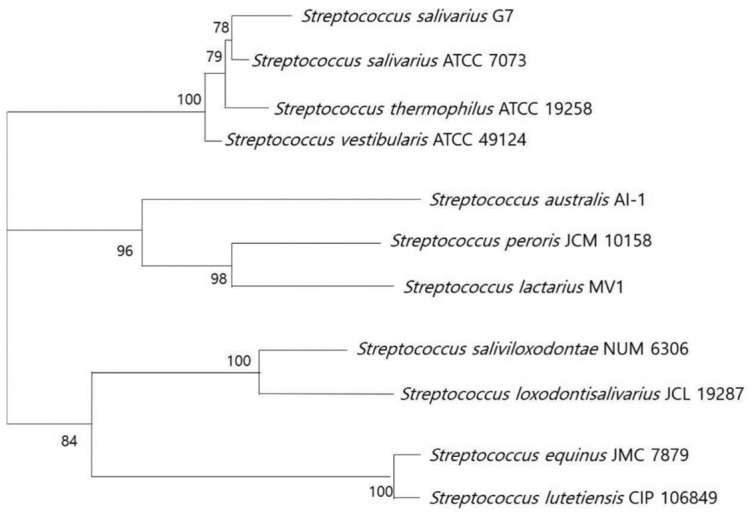
Phylogenetic reconstruction of *S. salivarius* G7. Phylogenetic trees were constructed using maximum likelihood methods. The numbers at nodes indicate the percentage occurrences among 1000 bootstrap values.

The *S. salivarius* G7 genome was subjected to whole-genome shotgun sequencing with Illumina and PacBio technology. About 4049 million reads were input into the Platanus-allee genome assembler, with a 2.14 Mb genome sequence generated. The assembly consisted of 8 contigs with an N50 length of 1,305,693 bp. The overall GC content of the *S. salivarius* G7 genome was 39.8%. When the nucleotide sequence of the complete 16S rRNA gene of *S. salivarius* G7 was compared with the DNA sequence database using the NCBI BLAST 2.15.0 program, the closest matches for the DNA sequence of *S. salivarius* G7 were *S. salivarius* (99.86 homologous) and *Streptococcus thermophilus* (99.68% homologous). Phylogenetic trees were constructed using the 16S rRNA sequences (Supplementary Table S1) and maximum likelihood methods (Figure 1). In the analysis, potential antibiotic resistance genes in the ORFs of the *S. salivarius* G7 genome were detected (Supplementary Table S2). Also, five bacteriocins (*slv*W, *slv*Y, *slv*Z, *psn*I, and *psn*L) were detected in three loci (Supplementary Table S3).

### 3.2. Metabolic Profiles of S. salivarius G7

The carbohydrate fermentation profile of *S. salivarius* G7 was analyzed using the API 50CH test kit. *S. salivarius* G7 was found to ferment 16 carbohydrates (Supplementary Table S4). Comparing *S. salivarius* K12, there were no differences in terms of fermentation, but there were fermentation differences L-arabinose, D-melibiose, glycogen, gentiobiose, amygdalin, and D-tagatose observed when compared to *S. salivarius* M18. Also, the G7 strain was confirmed as *S. salivarius* through the API 20 strep test (Table 1).

### 3.3. Hemolytic Activity and D-Lactate Production

The hemolytic activity of *S. salivarius* G7 was investigated using a sheep blood agar plate. The zone surrounding *S. salivarius* G7 developed a dark green color, indicating alpha hemolysis (Supplementary Figure S1). The positive control of β-hemolysis is *Streptococcus pyogenes*, and it was grown on a medium that developed a complete hemolytic zone (yellow color) (Supplementary Figure S1).

When D-lactate accumulates in the blood of people with short bowel syndrome, it leads to a manifestation of D-lactic acidosis and encephalopathy. D-lactate levels in the fresh and conditioned media of 12.12 ± 0.35 nmol/μL and 4.47 ± 0.60 nmol/μL, respectively, were detected. As the final outcome, *S. salivarius* G7 was found not to produce D-lactate (Table 2).

### 3.4. Antibiotic Susceptibility Assay

The antibiotic resistance of *S. salivarius* G7 was investigated using E-test strips that contained ampicillin, chloramphenicol, clindamycin, erythromycin, gentamycin, kanamycin, streptomycin, tetracycline, and vancomycin. *S. salivarius* G7’s resistance to antibiotics was evaluated, and its susceptibility was found to be below the cut-off value provided in the EFSA guidelines (Table 3).

### 3.5. Cytotoxicity

**Figure 2:**
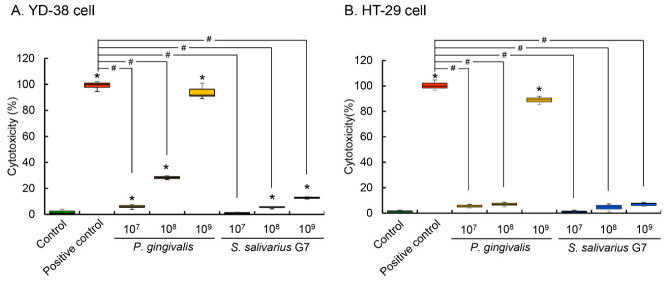
Cytotoxicity to oral and intestinal cells. Oral cell line (YD-38) (**A**) and colon cell line (HT-29) (**B**) were treated with *S. salivarius* G7 and pathogenic bacteria such as *P. gingivalis* and *E. coli*. Also, a positive control was used: Triton X-100. After incubating, the levels of lactate dehydrogenase (LDH) were measured. The average O.D value of the positive control group was set to 100%, and the O.D values of other groups were converted to % values. Data are represented as the median (horizontal lines), interquartile range (boxes), and full ranges (whiskers). Asterix (*) indicates statistically significant difference compared to control group (*p* < 0.05), and sharp (#) indicates statistically significant difference compared to positive control group (*p* < 0.05).

The cytotoxicity of *S. salivarius* G7 was investigated by comparing the expression levels of lactate dehydrogenase using *P. gingivalis* and *E. coli* as comparative controls for YD-38 and HT-29, respectively. Compared with the positive control group, 10^7^ and 10^9^ CFU of *P. gingivalis* showed lactate dehydrogenase expression at levels of 20.31 ± 6.37% and 89.97 ± 3.45%, respectively, while 10^7^ and 10^9^ CFU of *S. salivarius* G7 showed expression levels of 1.76 ± 2.94% and 42.93 ± 2.22%, respectively (Figure 2A). Also, for HT-29 cells, 10^7^ and 10^9^ CFU of *E. coli* showed lactate dehydrogenase expression levels of 5.70 ± 0.49% and 88.15 ± 1.85%, respectively, while 10^7^ and 10^9^ CFU of *S. salivarius* G7 showed lactate dehydrogenase expression levels of 0.81 ± 0.98% and 7.583 ± 0.93%, respectively (Figure 2B).

### 3.6. Acidogenicity

**Figure 3:**
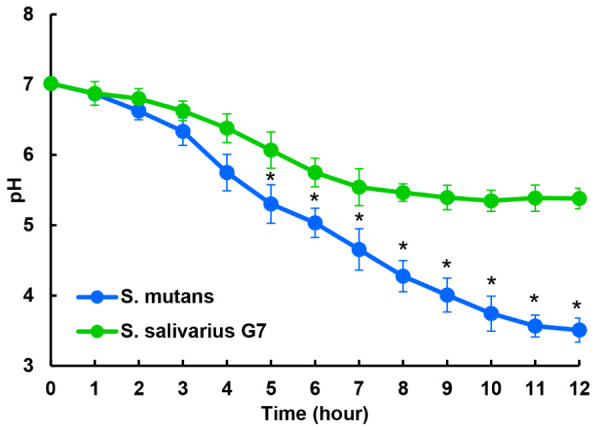
Acidogenicity of *S. salivarius* G7. *S. salivarius* G7 and *S. mutans* were inoculated into TSB including 2% sucrose and then incubated. The bacterial suspension was measured pH level using pH meter. Data are represented as the mean and standard deviation. Asterix (*) indicates statistically significant difference compared to *S. mutans* at each hour (*p* < 0.05).

In addition to cytotoxicity, strong acid production may induce dental caries, so this was also evaluated for the oral probiotics. Compared to the acid production of caries-related *S. mutans*, *S. salivarius* showed a mildly lower pH curve (Figure 3). When *S. mutans* and *S. salivarius* G7 were cultured for 12 h, the final pH values were 3.50 ± 0.25 and 5.38 ± 0.14, respectively.

### 3.7. Antimicrobial Activity Against Oral Pathogens

**Figure 4:**
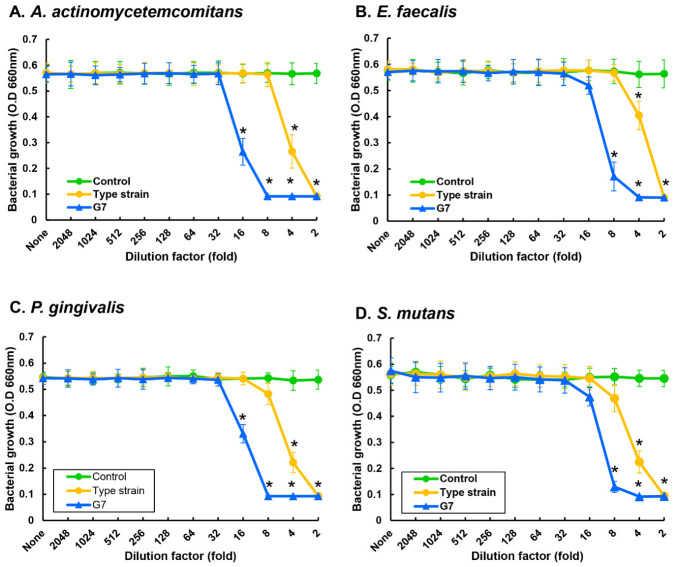
Antimicrobial activity of *S. salivarius* G7. *S. salivarius* conditioned medium (SCM) was collected and serially diluted in 96-well plate. *A. actinomycetemcomitans* (**A**), *E. faecalis* (**B**), *P. gingivalis* (**C**), and *S. mutans* (**D**) were inoculated into the prepared plates. The growth of bacteria was measured by optical density using a spectrophotometer. Asterix (*) indicates statistically significant difference compared to control group at each dilution factor (*p* < 0.05).

To investigate the ability of *S. salivarius* G7 to produce bacteriocin, its antimicrobial activity was examined using oral pathogens. The spent culture medium of *S. salivarius* showed antibacterial activity against oral pathogens, including *A. actinomycetemcomitans, E. faecalis, P. gingivalis*, and *S. mutans* (Figure 4). Compared to the antimicrobial activity of the *S. salivarius* type strain, *S. salivarius* G7 showed stronger activity against oral pathogens. The growth of *A. actinomycetemcomitans* and *P. gingivalis*, *E. faecalis*, and *S. mutans* was significantly inhibited in the media containing *S. salivarius* G7 at dilutions exceeding 16-fold of spent culture medium (Figure 4A,C). The growth of *E. faecalis and S. mutans* was significantly reduced in the media containing *S. salivarius* G7 at dilutions exceeding 8-fold of spent culture medium (Figure 4B,D).

### 3.8. Effect of S. salivarius G7 on Cariogenic Biofilm

**Figure 5:**
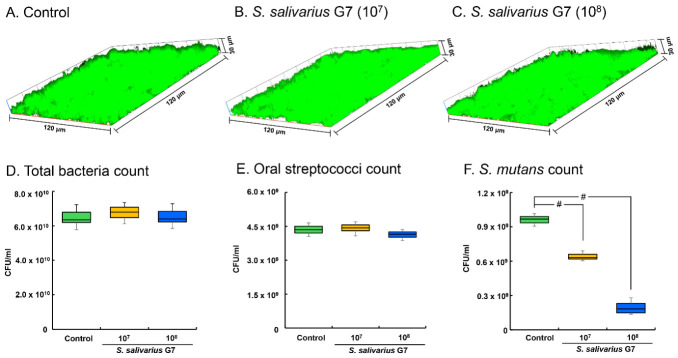
Inhibitory effect of *S. salivarius* G7 on formation of cariogenic biofilm. Collected bacteria from unstimulated saliva formed biofilm with *S. mutans* in the presence or the absence of *S. salivarius* G7. The biofilm formation was observed by CLSM after staining with the live/dead staining kit (**A**–**C**). Also, the bacterial composition in the biofilm was analyzed by bacteria incubation with BHI agar ((**D**), total bacteria), Mitis-salivarius agar ((**E**), Oral streptococci), and Mitis-salivarius bacitracin agar ((**F**), *S. mutans*). Control group is saliva biofilm including *S. mutans* as a cariogenic biofilm. Data are represented as the median (horizontal lines), interquartile range (boxes), and full ranges (whiskers). Sharp (#) indicates statistically significant difference compared to control group (*p* < 0.05).

*S. mutans* was added to the biofilm created by salivary bacteria to form a cariogenic biofilm. This was used as a control group, and compared to the group with added *S. salivarius* G7, no significant difference was observed (Figure 5A–C). In the total bacteria and oral streptococci culture in BHI and MS media, no significant difference was observed between the control group and that treated with *S. salivarius* G7 (Figure 5D,E), but in the selective culture of *S. mutans* using MSB medium, the treated *S. salivarius* G7 showed a significant decrease compared to the control group (Figure 5F). Additionally, when the ratio of *S. mutans* to oral streptococci was calculated, *S. mutans* was found to decrease depending on the concentration of *S. salivarius* G7.

## 4. Discussion

Transitional probiotic strains have demonstrated safety through long-term use, whereas recently developed probiotics are required to provide rigorous evidence of safety to obtain regulatory approval. The WHO/FAO have proposed development guidelines for their evaluation [2]. In this study, *S. salivarius* G7, a newly identified probiotic candidate, was assessed for probiotic suitability and its potential benefits for oral health.

The first step in the safety assessment of a candidate probiotic strain is to establish precise taxonomic identification [23]. To achieve this, whole-genome sequencing and metabolic profiling were performed. *S. salivarius* G7 was isolated from the oral cavity of a healthy adult male, and its taxonomic identity was confirmed through 16S rRNA gene sequencing and multi-gene phylogenetic reconstruction. In addition, the metabolic profile of the G7 strain was verified using API 50 CH and SPI 20 Strep analysis. Both genetic and metabolic assessments confirmed that *S. salivarius* G7 lacks characteristics that pose a significant risk to human health.

Subsequently, additional safety evaluations were conducted, including assessments of antibiotic resistance, hemolytic activity, cytotoxicity, and D-lactate production. European Food Safety Authority (EFSA) guidelines were followed for susceptibility testing [22]. With the increasing global prevalence of antibiotic-resistant bacteria, antibiotic resistance and the horizontal transfer of resistance genes in microorganisms are an important crisis facing the public health community [24]. Therefore, probiotics used as dietary supplements must be both susceptible to medically relevant antibiotics and not harbor any transferable antibiotic resistance genes. The MICs of *S. salivarius* G7 for a broad range of antibiotic classes were below the breakpoints established by EFSA. As commonly observed in oral streptococci, *S. salivarius* G7 exhibited α-hemolysis (partial hemolysis), which is a normal feature of oral commensals. For cytotoxicity testing, oral cell lines and colon epithelial cell lines were used to enhance accuracy, and oral and colon pathogens were used as positive controls. *S. salivarius* G7 is a probiotic for oral health, but it can enter the gut upon ingestion. Therefore, to investigate its effects on the gut environment, cytotoxicity was also evaluated on HT-29 as a colon cancer cell line. The G7 strain showed significantly lower lactate dehydrogenase levels compared to the pathogens, indicating that it is likely non-cytotoxic and safe for human use. Finally, D-lactate production was examined, as D-lactate accumulation in patients with short bowel syndrome or intestinal failure can lead to D-lactic acidosis and encephalopathy [2]. *S. salivarius* G7 did not produce D-lactate. Although the *Streptococcus* genus is known to ferment lactose primarily to L-lactate, additional experiments were conducted to confirm that *S. salivarius* G7 does not produce D-lactate.

Current probiotic guidelines primarily focus on safety and benefits for gastrointestinal health and do not address site-specific characteristics, such as those of the oral cavity [2,3]. The oral cavity is a unique environment containing both hard and soft tissues, including mucosal soft tissues and hard tissues such as teeth. In particular, enamel demineralization of tooth enamel begins below pH values of 5.5, which could lead to the induction of dental caries [25]. Therefore, the acidogenicity of *S. salivarius* G7 was evaluated. The cultured media of *S. salivarius* G7 and *S. mutans* reached pH values below 5.5 after 8 and 5 h, respectively. When fully cultured, the mean pH values were 5.38 for *S. salivarius* G7 and 3.50 for *S. mutans*. These findings suggest that *S. salivarius* G7 is unlikely to contribute to dental caries formation. Diseases involving soft tissues in the oral cavity, such as periodontitis and apical periodontitis (pulpitis), were also examined. *S. salivarius* G7 inhibited the growth of several key pathogens associated with these diseases, including *A. actinomycetemcomitans*, *E. faecalis*, and *P. gingivalis*.

The oral cavity hosts more than 700 microbial species, and the “extended ecological plaque hypothesis” has been proposed to explain the pathogenesis of oral diseases [26]. This hypothesis suggests that dental caries is not caused by the presence of specific bacteria, but rather through a process of “ecological change” in which the bacterial composition changes due to the acidification of the entire oral ecosystem. The initial microbial ecosystem of a healthy tooth surface consists primarily of non-mutans streptococci and actinomycetes. Because non-mutans streptococci and actinomycetes possess lower acid-producing capacity and acid resistance compared to mutans streptococci, their weak acid production maintains a balance between tooth demineralization and remineralization through “mild and infrequent acidification” of the surrounding tooth environment (dynamic stability phase). When the plaque environment becomes acidic due to sucrose intake or decreased saliva secretion, the growth of mutans streptococci increases, and a low-pH environment eventually causes mutans streptococci to become the dominant species. The persistence of this acidic environment subsequently leads to the development of dental caries. In the present study, the finding that *S. salivarius* G7 reduced the level of S. mutans without suppressing overall biofilm formation suggests that *S. salivarius* G7 helps maintain an ecological environment dominated by non-mutans streptococci. This finding suggests the possibility of inhibiting the proliferation and nutrient competitiveness of mutans streptococci, which thrives in low-pH environments, by preventing ecosystem changes toward acidification. However, this result has limitations in that it may differ from general oral biofilms, as the biofilm was formed using salivary bacteria collected from specific regions, specific age groups, and small populations. These results indicate that *S. salivarius* G7 may promote a shift to a mild acidification state as a healthy condition.

## 5. Conclusions

*S. salivarius* G7 met all safety assessment criteria required by current probiotic guidelines, including those for genetic characterization, antibiotic susceptibility, and cytotoxicity. Moreover, *S. salivarius* G7 exhibited properties beneficial for oral health, such as low acidogenicity, antibacterial activity against oral pathogens, and the ability to support microbial homeostasis. These findings suggest that *S. salivarius* G7 may be a safe probiotic candidate for oral health applications.

## Figures and Tables

**Table 1:** Carbohydrate fermentation and metabolic profile of *S. salivarius* G7 were analyzed with API 20 strep strips.

Enzyme Reaction	Activity
Acetoin production	+
Hippuric acid hydrolysis	−
β-glucoside hydrolysis	+
Pyrrolidonyl arylamidase	−
α-galactosidase	+
β-glucuronidase	−
β-Galactosidase	+
Alkaline phosphatase	−
Leucine aminopeptidase	+
Arginine dihydrolase	−
D-ribose	−
L-arabinose	−
D-mannitol	−
D-sorbitol	−
D-lactose	+
D-trehalose	+
Inulin	+
D-raffinose	+
Starch	−
Glycogen	−

**Table 2:** D-lactate production of S. salivarius G7.

Sample	nmol/μL
Fresh medium	12.12 ± 0.35
Conditioned medium	4.47 ± 0.60

**Table 3:** Susceptibility of *S. salivarius* G7 to antibiotics.

	Streptococcus G7 (mg/L)	Cut-Off Value of ESFA
Ampicillin	0.094	2
Chloramphenicol	1.5	4
Clindamycin	0.064	2
Erythromycin	0.25	2
Gentamicin	8	32
Kanamycin	32	64
Streptomycin	48	64
Tetracycline	0.75	4
Vancomycin	0.38	4

## Data Availability

The transcriptome raw reads with Bio-project Accession no. MT459250.1 is available in NCBI GenBank.

## Supplementary Materials

The following supporting information can be downloaded at: https://www.mdpi.com/article/10.3390/microorganisms14061234/s1, Figure S1: Hemolytic property of *S. salivarius* G7; Table S1: 16s rRNA sequence of *S. salivarius* G7; Table S2: Resistance gene identification of *S. salivarius* G7; Table S3: Identified bacteriocins of *S. salivarius* G7; Table S4: Carbohydrate fermentation and metabolic profile of *S. salivarius* G7 was analyzed with API50CHL and API 20 strep strips.
