# Supplementary material for: Newly Emerging Streptococcus salivarius G7 as a Probiotic Candidate for Oral Health
Source: Microorganisms. 2026 May 30;14(6):1234. doi: 10.3390/microorganisms14061234 (PMC13302888; doi:10.3390/microorganisms14061234)
Supplement: Supplementary file 1 [file microorganisms-14-01234-s001.zip › supplemantary table S1 (DNA sequencing).pdf]

Supplementary Table S1. 16s rRNA sequence of S. salivarius G7.

GACGAACGCT GGCGGCGTGC CTAATACATG CAAGTAGAAC GCTGAAGAGA GGAGCTTGCT
CTTCTTGGAT GAGTTGCGAA CGGGTGAGTA ACGCGTAGGT AACCTGCCTT GTAGCGGGGG
ATAACTATTG GAAACGATAG CTAATACCGC ATAACAATGG ATGACCCATG TCATTTATTT
GAAAGGGGCA AATGCTCCAC TACAAGATGG ACCTGCGTTG TATTAGCTAG TAGGTGAGGT
AACGGCTCAC CTAGGCGACG ATACATAGCC GACCTGAGAG GGTGATCGGC CACACTGGGA
CTGAGACACG GCCCAGACTC CTACGGGAGG CAGCAGTAGG GAATCTTCGG CAATGGGGGC
AACCCTGACC GAGCAACGCC GCGTGAGTGA AGAAGGTTTT CGGATCGTAA AGCTCTGTTG
TAAGTCAAGA ACGAGTGTGA GAGTGGAAAG TTCACACTGT GACGGTAGCT TACCAGAAAG
GGACGGCTAA CTACGTGCCA GCAGCCGCGG TAATACGTAG GTCCCGAGCG TTGTCCGGAT
TTATTGGGCG TAAAGCGAGC GCAGGCGGTT TGATAAGTCT GAAGTTAAAG GCTGTGGCTC
AACCATAGTT CGCTTTGGAA ACTGTCAAAC TTGAGTGCAG AAGGGGAGAG TGGAATTCCA
TGTGTAGCGG TGAAATGCGT AGATATATGG AGGAACACCG GTGGCGAAAG CGGCTCTCTG
GTCTGTAACT GACGCTGAGG CTCGAAAGCG TGGGGAGCGA ACAGGATTAG ATACCCTGGT
AGTCCACGCC GTAAACGATG AGTGCTAGGT GTTGGATCCT TTCCGGGATT CAGTGCCGCA
GCTAACGCAT TAAGCACTCC GCCTGGGGAG TACGACCGCA AGGTTGAAAC TCAAAGGAAT
TGACGGGGGC CCGCACAAGC GGTGGAGCAT GTGGTTTAAT TCGAAGCAAC GCGAAGAACC
TTACCAGGTC TTGACATCCC GATGCTATTT CTAGAGATAG AAAGTTACTT CGGTACATCG
GTGACAGGTG GTGCATGGTT GTCGTCAGCT CGTGTCGTGA GATGTTGGGT TAAGTCCCGC
AACGAGCGCA ACCCCTATTG TTAGTTGCCA TCATTCAGTT GGGCACTCTA GCGAGACTGC
CGGTAATAAA CCGGAGGAAG GTGGGGATGA CGTCAAATCA TCATGCCCCT TATGACCTGG
GCTACACACG TGCTACAATG GTTGGTACAA CGAGTTGCGA GTCGGTGACG GCAAGCTAAT
CTCTTAAAGC CAATCTCAGT TCGGATTGTA GGCTGCAACT CGCCTACATG AAGTCGGAAT
CGCTAGTAAT CGCGGATCAG CACGCCGCGG TGAATACGTT CCCGGGCCTT GTACACACCG
CCCGTCACAC CACGAGAGTT TGTAACACCC GAAGTCGGTG AGGTAACCTT TTGGAGCCAG
CCGCCTAAGG TGGGATAGAT GATTGGGGTG
