# Supplementary material for: Newly Emerging Streptococcus salivarius G7 as a Probiotic Candidate for Oral Health
Source: Microorganisms. 2026 May 30;14(6):1234. doi: 10.3390/microorganisms14061234 (PMC13302888; doi:10.3390/microorganisms14061234)
Supplement: Supplementary file 1 [file microorganisms-14-01234-s001.zip › supplementary table S3 (identified bacteriocin).pdf]

Supplemented Table S3. Identified bacteriocins of *S. salivarius* G7

Name	DNA sequences	Protein sequences	gene Location
slvZ	ATGACTAAGACCATTAACAATCGTAAAAACATGACTACTCAAGAACTTGAAA CTGTATCAGGTGGAGTGGTTCCTTGGGCCGCTATTTCTGTTGGCATTGCTGCT GCAAAACTAACTTATGACCTTAGCTATGCTGCAGGTAAGTCTTTCTATAACCT CACCCACTAA	MTKTINNRKNMTTQELETVSGGVVPWAAIS VGIAAAKLTYDLSYAAGKSFYNLTH	235,700
slvY	ATGACAATGATTAACAAAGAAATGAAGGCAGCTGACCTAGCCTCAGTAACA GGAGGCGGATGGAAGACTAACCTTGCCATTGGAGGGCTCTGCCTAGCTTCA GGACCTATTGGAACTATGGTATGCCTTGGAGCCTACAATGGCTACATGGACT CTGCGAGATAA	MTMINKEMKAADLASVTGGGWKTNLAIGG LCLASGPIGTMVCLGAYNGYMDSAR*	235,516
slvW	ATGCGAACTAAGGTATATGGTGAAGAGCTCAATGCTGAAAGCTTAGAAAAT GTTACAGGGGGTGGTTTTGTATCTAAACCTCAAACCTTACCTGAACGATTGG GTTGGAACAAATGGTGGTTGAAAAGAAGACCTCCTTATGGCGATTAG	MRTKVYGEELNAESLENVTGGGFVSKPQTLP ERLGWNKWWLKRRPPYGD*	789,444
psnI	ATGACAACACAAATCATTAACAATTTTAATTCACTTAATTCCGAAGATCTTTC TATTATCGAAGGTGGGGGTGTGATTGGTTGTGTAGCTGGGACTGCTGGATCT GCTGGGCTTGGATTTTTGACTGGAACATCAGTTGGAACTGTTACATTTCCAAT TGTTGGGACTGTCTCAGGTGGAGCATTTGGGGCATGGTCAGGAGCTGGCCTT GGAATGGCTACATTTTGCGGAGTTTAG	MTTQIINNFNSLNSEDLSIIEGGGVIGCVAGT AGSAGLGFLTGTSVGTVTFPIVGTVSGGAFG AWSGAGLGMATFCGV*	856,089
psnL	ATGACAACACAAACAATGAACACTTTTGAAACACTCGATCTCGAGACACTTG CAAACGTTGAAGGTGGCGGATGGGTTAAGTGTTATGCCGGCACAATTGGCTC TGCTCTAGTAGGATCAGCAGGAGGCCCGGTAGGTTACTGGGGAGGAGCTTTA GTTGGCTATGCTACATTCTGCTAA	MTTQTMNTFETLDLETLANVEGGGWVKCYA GTIGSALVGSAGGPVGYWGGALVGYATFC*	864,717
