# Supplementary material for: Newly Emerging Streptococcus salivarius G7 as a Probiotic Candidate for Oral Health
Source: Microorganisms. 2026 May 30;14(6):1234. doi: 10.3390/microorganisms14061234 (PMC13302888; doi:10.3390/microorganisms14061234)
Supplement: Supplementary file 1 [file microorganisms-14-01234-s001.zip › supplementary table S4 (metaboliic characters).pdf]

Supplementary Table S4. Carbohydrate fermentation and metabolic profile of *S. salivarius* G7 was analyzed with API50CHL and API 20 strep strips.

API 50 CHL			
**Substrate**	**Fermentation**	**Substrate**	**Fermentation**
Glycerol	-	Salicin	+
Erythritol	-	Celiobiose	+
D-arabinose	-	Maltose	+
L-arabinose	-	Lactose	+
D-ribose	-	Melibiose	-
D-xylose	-	Sucrose	+
L-xylose	-	Trehalose	+
D-adonitol	-	Inulin	+
Methyl-β-D-xylopyranoside	-	Melezitose	-
D-galactose	+	D-raffinose	+
D-glucose	+	Starch	-
D-fructose	+	Glycogen	-
D-mannose	+	Xylitol	-
L-sorbose	-	Gentiobiose	-
L-rhamnose	-	D-turanose	-
Dulcitol	-	D-lyxose	-
Inositol	-	D-tagatose	+
D-mannitol	-	D-fucose	-
D-sorbitol	-	L-fucose	-
α-Methyl-D-mannopyranosdie	-	D-arabitol	-
α-Methyl-D-glucoside	-	L-arabitol	-
N-acethyl-glucosamine	+	Gluconate	-
Amygdalin	-	Potassium 2-ketogluconate	-
Arbutin	+	Potassium 5-ketogluconate	-
Esculin	+		
API 20 Strep			
**Enzyme reaction**	**Activity**	**Enzyme reaction**	**Activity**
Acetoin production	+	D-ribose	-
Hippuric acid hydrolysis	-	L-arabinose	-
β-glucoside hydrolysis	+	D-mannitol	-
Pyrrolidonyl arylamidase	-	D-sorbitol	-
α-galactosidase	+	D-lactose	+
β-glucuronidase	-	D-trehalose	+
β-Galactosidase	+	Inulin	+
Alkaline phosphatase	-	D-raffinose	+
Leucine aminopeptidase	+	Starch	-
Arginine dihydrolase	-	Glycogen	-
