# Supplementary figures and images for: Newly Emerging Streptococcus salivarius G7 as a Probiotic Candidate for Oral Health
Source: Microorganisms. 2026 May 30;14(6):1234. doi: 10.3390/microorganisms14061234 (PMC13302888; doi:10.3390/microorganisms14061234)

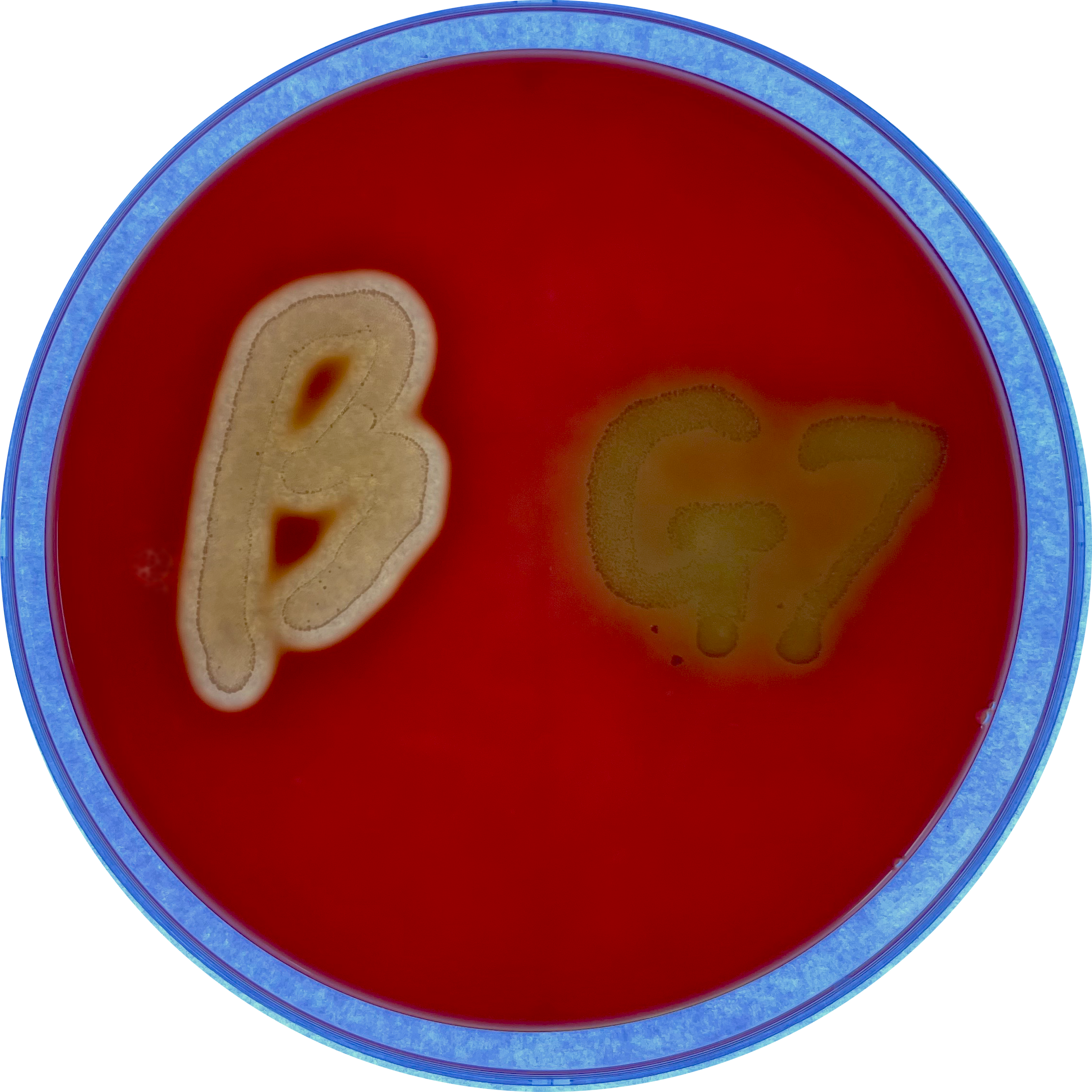

Supplement: Supplementary file 1 [file microorganisms-14-01234-s001.zip › supplementray figure 1.tif]
